# High-porosity Pt–CeO_2_ nanosponges as oxidation catalyst†

**DOI:** 10.1039/d4na00525b

**Published:** 2024-12-30

**Authors:** Simon Falkner, Carina B. Maliakkal, Mareike Liebertseder, Joachim Czechowsky, Maria Casapu, Jan-Dierk Grunwaldt, Christian Kübel, Claus Feldmann

**Affiliations:** a Institute of Inorganic Chemistry (IAC), Karlsruhe Institute of Technology (KIT) Engesserstraße 15 D-76131 Karlsruhe Germany claus.feldmann@kit.edu; b Institute of Nanotechnology (INT), Karlsruhe Nano Micro Facility (KNMFi), Karlsruhe Institute of Technology (KIT) Hermann-von-Helmholtz-Platz 1, 76344 Eggenstein-Leopoldshafen Germany; c Institute for Chemical Technology and Polymer Chemistry (ITCP), Karlsruhe Institute of Technology (KIT) Engesserstraße 20 76131 Karlsruhe Germany; d Institute of Catalysis Research and Technology (IKFT), Karlsruher Institute of Technology (KIT) Hermann-von-Helmholtz-Platz 1, 76344 Eggenstein-Leopoldshafen Germany

## Abstract

Pt–CeO_2_ nanosponges (1 wt% Pt) with high surface area (113 m^2^ g^−1^), high pore volume (0.08 cm^3^ g^−1^) and small-sized Pt nanoparticles (1.8 ± 0.4 nm) are prepared by thermal decomposition of a cerium oxalate precursor and examined for catalytic oxidation of CO, volatile organic compounds (VOCs), and NH_3_. The cerium oxalate precursor Ce_2_(C_2_O_4_)_3_·10H_2_O is prepared by aqueous precipitation from Ce(NO_3_)_3_·6H_2_O and K_2_C_2_O_4_·H_2_O and thermally converted to CeO_2_ nanosponges by heating in air. Optimal conditions for decomposition in terms of surface area and porosity are observed at 350 °C for 20 min. Finally, the CeO_2_ nanosponges are decorated with small-sized Pt nanoparticles, using a wet-chemical impregnation with Pt(ac)_2_ in methanol. Electron microscopy with tomography, electron spectroscopy and further methods (TG, XRD, FT-IR, sorption analysis) are used to characterize the catalyst composition and especially the structure and porosity of the Pt–CeO_2_ nanosponges as well as the uniform distribution of the Pt nanoparticles. The Pt–CeO_2_ nanosponges show good thermal stability (up to 400 °C) and, already as a new, non-optimized catalyst, promising activity for catalytic oxidation of CO, VOCs, NH_3_ as indicated by high activities in terms of low and stable light-out and light-off temperatures as well as a high selectivity to N_2_ (for NH_3_ oxidation) with >80% at 170–250 °C.

## Abstract 2

A highly porous Pt–CeO_2_ nanosponge catalyst is obtained by thermal transformation from a cerium oxalate precursor, showing good thermal stability and promising activity for catalytic oxidation of CO, VOCs, NH_3_ with high selectivity for N_2_.

## Graphical abstract

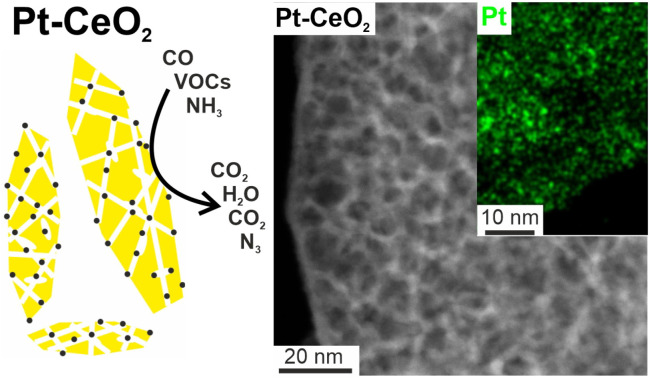

## Introduction

Ceria, in combination with a precious metal, is specifically interesting for oxidation catalysis as it combines several beneficial properties.^1^ Thus, the oxidation states Ce^3+^ and Ce^4+^ – specifically in the solid phases Ce_2_O_3_ and CeO_2_ – are of comparable stability,^2^ which promotes reversible redox reactions. Moreover, the structures of Ce_2_O_3_ (defect CaF_2_ type)^3^ and CeO_2_ (CaF_2_ type)^4^ are in close relation so that oxygen can be reversibly stored and released. With a Mars–van-Krevelen-like mechanism,^5^ oxygen at the interface of ceria and a noble metal (*e.g.*, Pt, Pd, Ru) can be provided for a catalytic oxidation of species (*e.g.*, CO, volatile organic compounds/VOCs, NH_3_) adsorbed on the precious metal.^6^ As a result, ceria is widely applied as a metal-oxide support in heterogeneous catalysis.^1^ Particularly important are emission control,^7^ reverse water–gas shift, electrocatalysis and fuel cells,^7^ direct synthesis of H_2_O_2_,^8^ or the detection of combustible gases.^9^

To promote heterogeneous catalytic reactions with high activity at low temperatures, ceria with high surface area and high porosity is required.^10^ On the one hand, this guarantees a sufficient contact between the solid metal-oxide support and the gaseous reactants. On the other hand, a uniform distribution of small-sized noble metal particles is possible over the large surface of the metal-oxide support. Due to its high oxide-ion conductivity, specifically at elevated temperature (>200 °C), however, ceria is also known for limited thermal stability. Thus, sintering due to the high oxide-ion mobility can result in a rapid decrease of surface area and porosity.^11^ Currently, the highest surface areas for ceria are reported with about 100 m^2^ g^−1^ for mesoporous mixtures of ceria and zirconia, using the latter to stabilize ceria.^12^ Higher surface areas of 170 m^2^ g^−1^ and 250 m^2^ g^−1^ were yet only reported for very small (1–5 nm) massive CeO_2_ nanoparticles.^13^ Despite a high surface area as such, ceria nanoparticles are not suitable for establishing porous 3D-networks and tend to form close-packed particle arrays. Furthermore, the respective high-surface-area CeO_2_ materials are not stable at higher temperature or were not even examined in regard of sintering and/or thermal shrinkage of the surface area.

Aiming at ceria metal-oxide supports with high surface area and porosity, we previously focused on ceria hollow nanospheres using either water droplets^14^ or sodium-chloride templates.^15^ The resulting ceria hollow nanospheres exhibited surface areas up to 210 m^2^ g^−1^ and pore volumes up to 0.08 cm^3^ g^−1^.^14,15^ The preparation and removal of the respective template, however, resulted in additional process steps for synthesis and purification. To facilitate the synthesis and specifically to reproducibly realize higher quantities, we here suggest a novel concept to obtain high-porosity ceria based on a cerium-oxalate precursor, which is thermally decomposed to ceria at low temperature (350 °C). After wet-chemical impregnation with Pt(ac)_2_, Pt–CeO_2_ nanosponges with high surface area (113 m^2^ g^−1^), high pore volume (0.08 cm^3^ g^−1^), small-sized Pt nanoparticles (1.8 ± 0.4 nm) and good temperature stability (up to 400 °C) were obtained and examined in regard to the catalytic oxidation of CO, VOCs (volatile organic compounds), and NH_3_.

## Results and discussion

### Synthesis of Ce_2_(C_2_O_4_)_3_ precursor particles

The need of high surface area and high porosity of CeO_2_ for catalysis was most often addressed either by hydrothermal, solvothermal or sol-gel processes,^16^ template-based techniques,^16,17^ or the preparation of nanostructured ceria with certain size and shape (*e.g.*, hollow spheres, nanorods, nanotubes).^18^ These approaches, on the one hand, require the presence of the respective porous host lattices and templates as well as a deep infiltration with CeO_2_ and/or a complete removal of the template. On the other hand, a sufficient thermal and chemical stability of the resulting material structure at the transient conditions occurring for catalysis (*e.g.*, variable temperature and pressure, presence of moisture and/or catalyst poisons) is mandatory. Concepts to transfer a suitable ceria precursor into high-surface porous ceria are rare by now. Most comparable is the so-called Pechini method^16,19^ with, for instance, Ce(NO_3_)_3_ converted to CeO_2_*via* combustion, with *e.g.*, glucose acting as a gelation agent.^20^ The temperature of reaction and the resulting surface area and porosity, however, are difficult to control *via* this approach.

**Fig. 1:**
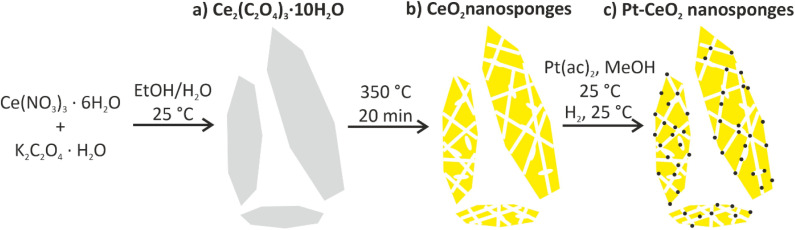
Scheme illustrating the synthesis of Pt–CeO_2_ nanosponges: (a) formation of rod- to platelet-shaped Ce_2_(C_2_O_4_)_3_·10H_2_O precursor particles, (b) thermal conversion of Ce_2_(C_2_O_4_)_3_·10H_2_O precursor particles to CeO_2_ nanosponges, (c) wet-chemical impregnation of the CeO_2_ nanosponges with a solution of Pt(ac)_2_ in methanol followed by Pt nanoparticle formation (at 25 °C).

Aiming at a suitable ceria precursor, which can be thermally transferred to CeO_2_ at moderate temperature, we here use cerium oxalate (Fig. 1a). Metal oxalates are generally well-known for low-temperature decomposition to metal oxides with release of CO/CO_2_. In this regard, cerium oxalate was yet only described to obtain nanocrystalline ceria with rod-type shape.^21^ Since Ce_2_(C_2_O_4_)_3_ is insoluble in water, the precursor particles can be obtained by simple aqueous precipitation. Accordingly, a solution of Ce(NO_3_)_3_·6H_2_O in ethanol was injected with a solution of K_2_C_2_O_4_·H_2_O in water. Thereafter, the formation of Ce_2_(C_2_O_4_)_3_ is indicated by the instantaneous nucleation of particles and the formation of a suspension. The resulting colourless solid was then separated by centrifugation and purified by repeated redispersion/centrifugation in/from water.

**Fig. 2:**
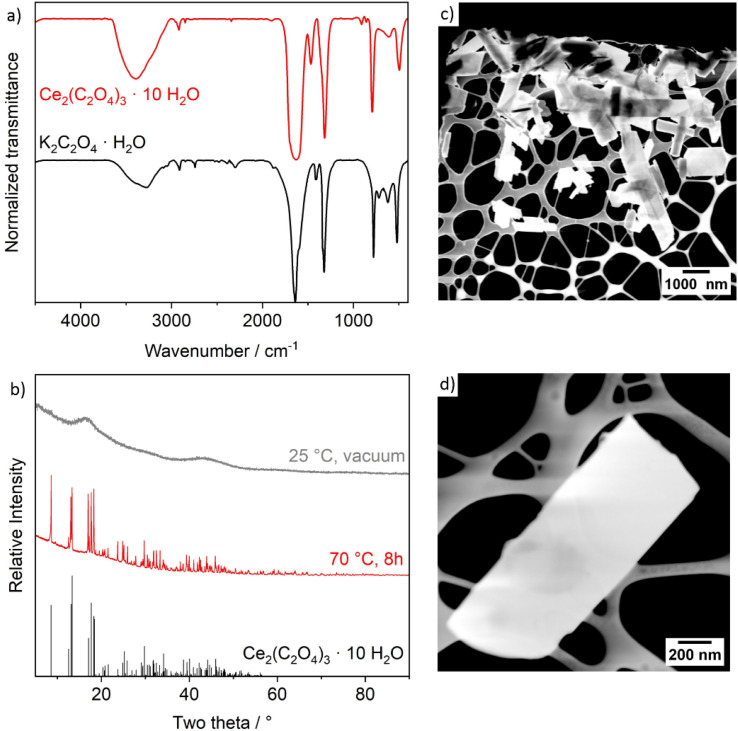
Characterization of the Ce_2_(C_2_O_4_)_3_·10H_2_O precursor particles: (a) FT-IR spectrum (K_2_C_2_O_4_·H_2_O as a reference), (b) XRD (Ce_2_(C_2_O_4_)_3_·10H_2_O as a reference, ICDD-No. C01-075-7101), (c) STEM overview image, (d) STEM detail image of a single precursor particle.

**Fig. 3:**
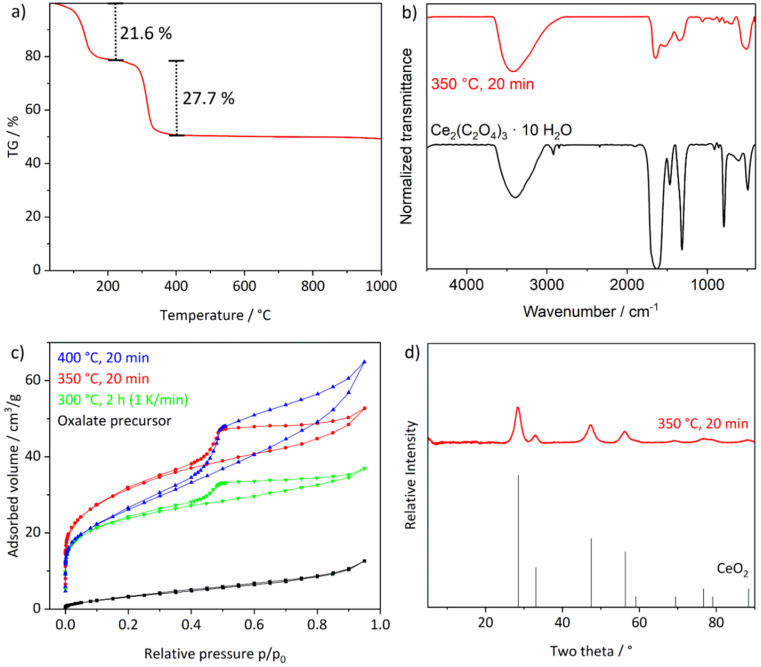
Characterization of the CeO_2_ nanosponges: (a) thermogravimetric analysis, (b) FT-IR spectra before and after thermal conversion, (c) volumetric sorption analysis after different thermal treatments, (d) XRD (CeO_2_ as a reference, ICDD-No. C00-034-0394).

The as-prepared Ce_2_(C_2_O_4_)_3_ precursor particles were characterized by Fourier-transform infrared (FT-IR) spectroscopy, X-ray powder diffraction (XRD), scanning transmission electron microscopy (STEM), and sorption analysis. FT-IR spectra of the Ce_2_(C_2_O_4_)_3_ precursor particles clearly evidences the presence of the characteristic vibrations of [C_2_O_4_]^2−^ (*ν*_as_(C=O): 1630, *ν*_s_(C=O): 1315, *δ*_as_(COO): 790, *δ*_s_(COO): 495 cm^−1^), which are also in accordance with K_2_C_2_O_4_·H_2_O as the starting material (Fig. 2a). In addition, *ν*(O–H) (3600–3000 cm^−1^) indicates the presence of water. After drying at room temperature in vacuum, XRD only shows non-specific, broad reflexes of a predominately amorphous compound (Fig. 2b). Subsequent to heating (70 °C, 8 h), the precursor particles become crystalline and can be clearly identified as Ce_2_(C_2_O_4_)_3_·10H_2_O. Scanning transmission electron microscopy (STEM) shows rod- to platelet-shaped particles with a length of 0.5–3.5 μm and a diameter of 200–650 nm (Fig. 2c). Detail STEM images indicate the precursor particles to be non-porous (Fig. 2d). Finally, sorption analysis with nitrogen as the sorbent and an analysis *via* the Brunauer–Emmett–Teller (BET) approach confirms the presence of a dense material with a surface area of 14 m^2^ g^−1^ and a pore volume of 0.015 cm^3^ g^−1^ only (Table 1, Fig. 3c and S1†).

**Table 1:** Surface area and pore volume of the Ce_2_(C_2_O_4_)_3_·10H_2_O precursor particles as well as of the CeO_2_ nanosponges depending on the conditions of thermal decomposition

Conditions for thermal decomposition	Surface area (m^2^ g^−1^)	Pore volume (cm^3^ g^−1^)
Ce_2_(C_2_O_4_)_3_·10H_2_O (as-prepared)	14	0.015
300 °C (2 h, 1 K min^−1^)	86	0.057
350 °C (20 min)	113	0.082
400 °C (20 min)	93	0.100

### Thermal decomposition to CeO_2_ nanosponges

For the thermal conversion of the Ce_2_(C_2_O_4_)_3_ precursor particles to porous CeO_2_ nanosponges (Fig. 1b), first of all, suitable conditions for the thermal decomposition need to be identified. Accordingly, thermogravimetry (TG) was conducted (Fig. 3a). Here, thermal decomposition is observed in two steps. First, a mass loss of 22% is observed at 30–210 °C, which is followed by a second mass loss at 210–400 °C with 28%. Hereof, the first step can be related to the evaporation of water (calculated: 24.9%). The second step relates to the loss of CO and CO_2_ (calculated: 27.6%) according to the following reaction: Ce_2_(C_2_O_4_)_3_ → 2CeO_2_ + 4CO + 2CO_2_. Based on this thermal behaviour, in principle, there are several options for decomposition: (i) a fast decomposition putting a powder sample into the already hot oven at the upper temperature limit (≥350 °C) or (ii) a slow decomposition with a ramp (1 K min^−1^) from room temperature to the lower temperature limit of decomposition (≤300 °C). Aiming at high surface area and porosity, both measures may have disadvantages. Fast heating may lead to a gas evolution being too fast and, thereby, destroying the thin walls of the intended nanosponges. Slow heating may cause extensive sintering due to the significantly longer time of the decomposition reaction. Therefore, we have examined different conditions (Table 1, Fig. 3c). With a surface area of 113 m^2^ g^−1^ and a pore volume of 0.082 cm^3^ g^−1^, fast heating (350 °C, 20 min) and fast decomposition turn out to be optimal (Table 1, Fig. 3c and S2a†). When exceeding 350 °C, the pore volume increases due to the formation of larger pores but with the surface area decreasing at the same time (Table 1, Fig. 3c). In regard of the pore diameter, predominately mesopores (≥25 Å and ≤ 45 Å) were observed (ESI Fig. S2b†). With these values, the CeO_2_ nanosponges are among the highest surface areas reported for CeO_2_. The highest specific surface area was yet reported with 100 m^2^ g^−1^ for mesoporous zirconia-ceria mixtures^12^ or with 170 m^2^ g^−1^ for very small (3–5 nm) massive CeO_2_ nanoparticles.^13^ Higher values of 250 m^2^ g^−1^ were – to the best of our knowledge – only reported for microemulsion-made CeO_2_ nanoparticles, which, however, results in very small amounts of material.^13^

**Fig. 4:**
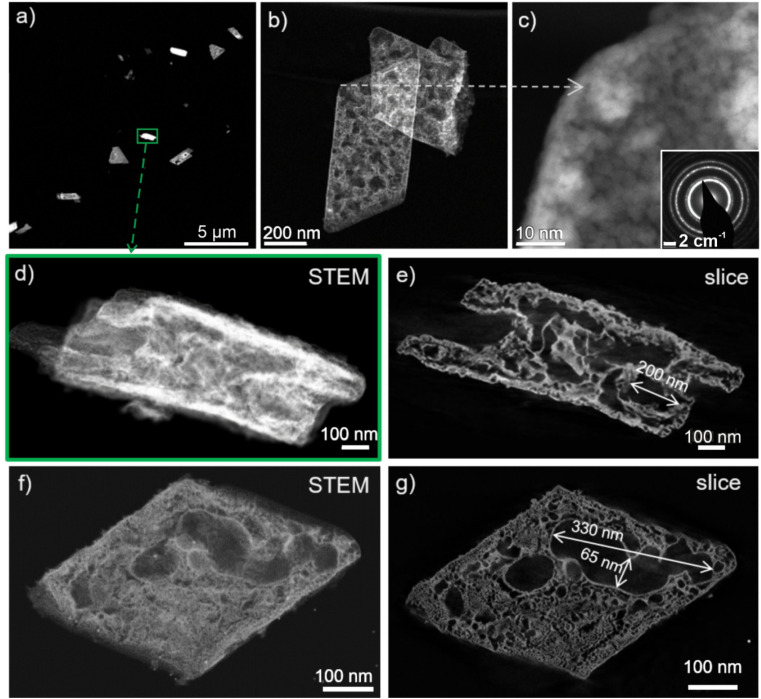
Electron microscopy and tomography of CeO_2_ nanosponges: (a–d and f) HAADF-STEM images at different magnification (inset in (c) showing the crystallinity, ESI Fig. S4†), (e and g) examples of Z-slices obtained from 3D electron tomographic reconstructions of nanosponges shown in (d) and (f) (for volume rendering and reconstruction of Z-slices see ESI Fig. S5 and Videos S1–S4†).

**Fig. 5:**
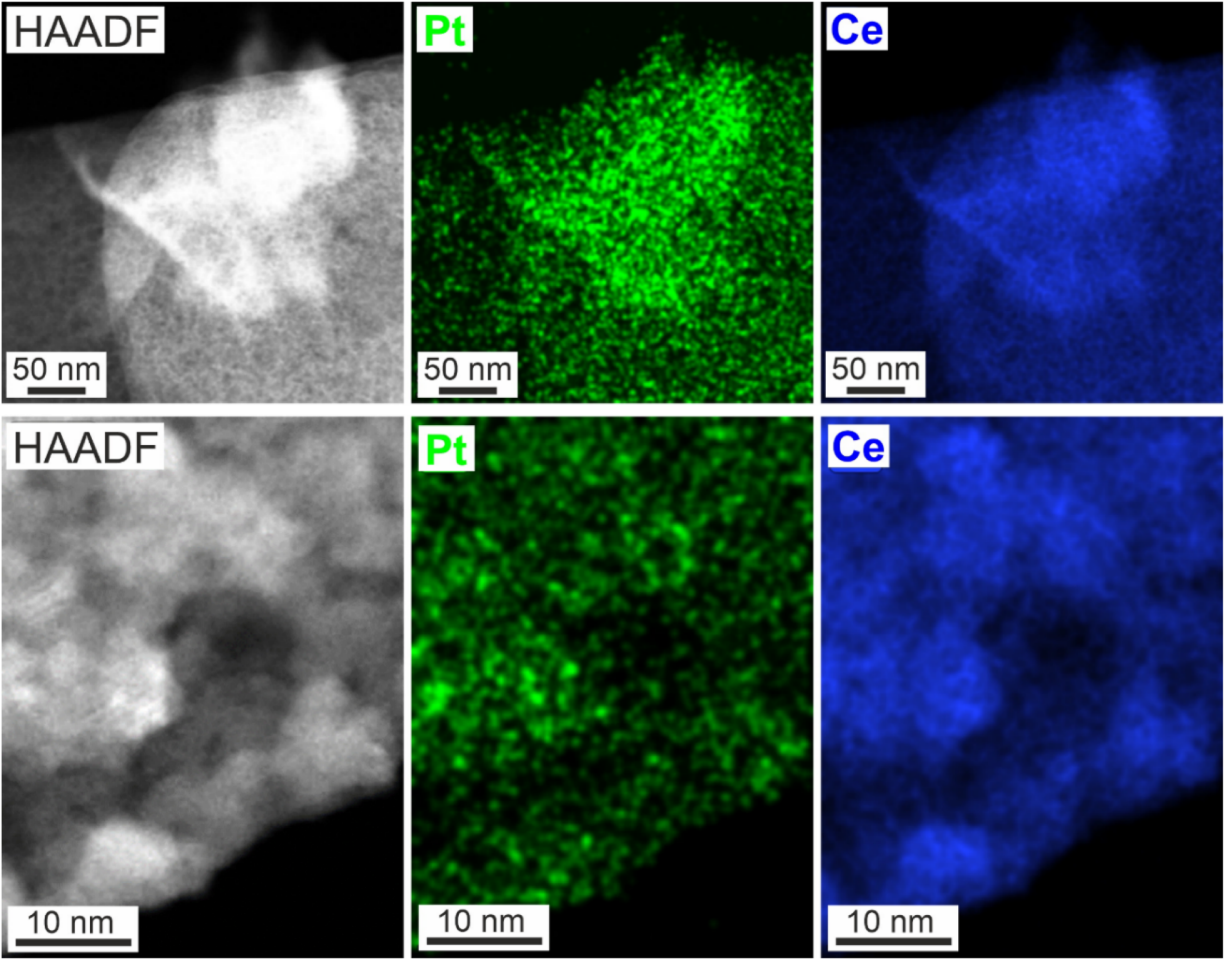
EDXS elemental maps of Pt on CeO_2_ nanosponges: STEM images of Pt–CeO_2_ nanosponges at different magnification and corresponding Pt and Ce elemental maps.

**Fig. 6:**
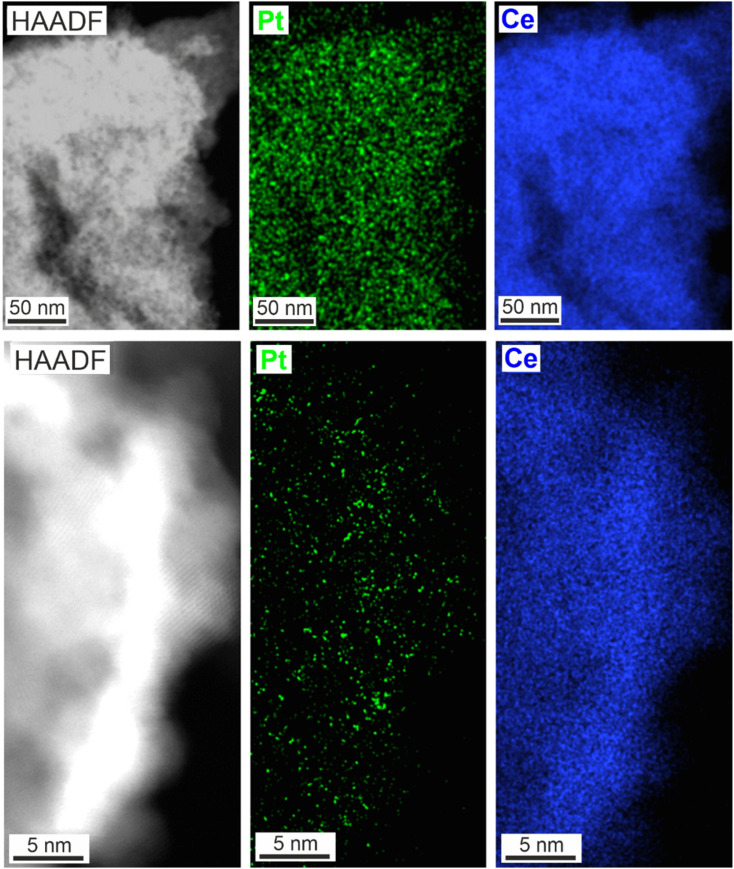
Thermal stability of the Pt–CeO_2_ nanosponges: STEM images of Pt–CeO_2_ nanosponges at different magnification and corresponding Pt and Ce EDXS elemental maps.

The conversion of the Ce_2_(C_2_O_4_)_3_·10H_2_O precursor particles is validated by XRD, which only shows the Bragg reflexes of CeO_2_ (Fig. 3d). FT-IR spectra only exhibit very weak vibrations related to remains of oxalate (Fig. 3b). The width of the Bragg reflexes already points to the presence of small crystallite sizes (Fig. 3d). Based on the Scherrer approach, a mean crystallite size of 4.5 nm can be deduced. In fact, this is in good agreement with the mean wall thickness of 3.5 ± 0.5 nm observed for the CeO_2_ nanosponges on TEM images (Fig. 4, 5 and 6). On a larger scale, STEM images still show a rod- to platelet-type shape with a length of 0.5–3.5 μm and a diameter of 200–650 nm of the Ce_2_(C_2_O_4_)_3_·10H_2_O precursor particles (Fig. 4). High-resolution STEM images and electron diffraction confirm the conversion of the dense precursor nanoparticles to high-surface-area and highly porous CeO_2_ nanosponges (Fig. 4b–g). STEM-based tomography reconstructions of the 3D structure further confirm the high porosity with a bimodal 3-dimensional network of interconnected large (up to 300 nm) and small pores (starting at 1–3 nm) (Fig. 4; for volume rendering and reconstruction of Z-slices see ESI Videos S1–S4†). Aiming at a catalyst material, such an interconnected fractal porous structure is beneficial as a fast gas-phase transport is possible *via* larger pores to reactive sites in smaller pores without the need of high pressure gradients.

### Decoration of CeO_2_ nanosponges with Pt nanoparticles

After the formation of the CeO_2_ nanosponges, the highly porous structure was impregnated with Pt precursors (1 wt% Pt) *via* a wet-chemical deposition process (Fig. 1c). To this concern, a solution of Pt(ac)_2_ in methanol was slowly dropped on a powder sample of the CeO_2_ nanosponges. Due to the low surface tension of methanol, the Pt(ac)_2_ solution was instantaneously distributed over the support surface due to capillary forces. Thereafter, the as-deposited Pt(ac)_2_ was reduced by reducing gas (N_2_ : H_2_ = 10 : 90) already at room temperature (25 °C). Due to the fine dispersion of Pt(ac)_2_ over the nanosponge surface prior to the reduction, a homogeneous distribution of small Pt nanoparticles is achieved. Accordingly, a uniform distribution of small Pt particles occurs with 1 wt% of Pt and a particle size of 1.0–2.5 nm (Fig. 5) and a mean size of 1.8 ± 0.4 nm (ESI Fig. S3 and S6†). This wet-chemical process with Pt(ac)_2_ is preferred here over the more often used aqueous solutions of platinum chloride or platinum nitrate^22^ as the latter require certain heating for reduction (100–300 °C), which promotes particle growth and leads to larger Pt nanoparticles than obtained here by reduction at room temperature.

STEM images display the uniform size distribution of the Pt nanoparticles all over the inner surface of the Pt–CeO_2_ nanosponges with the bright spots indicating the presence of high-density Pt nanoparticles (Fig. 5a), which was confirmed by energy-dispersive X-ray spectroscopy (EDXS) elemental mapping (Fig. 5b). Beside high surface area and porosity of the CeO_2_ nanosponges and a small size of the Pt nanoparticles, a sufficient thermal stability of the Pt–CeO_2_ catalyst system is required for catalytic application. This includes the sintering stability of the cerium-oxide support as well as the size stability of the Pt nanoparticles. As the CeO_2_ nanosponges were prepared by thermal decomposition of Ce_2_(C_2_O_4_)_3_·10H_2_O at 350 °C, a sufficient thermal stability at least up to this temperature can be expected.

The thermal stability of the Pt–CeO_2_ nanosponges was examined by XRD, sorption analysis, and TEM up to a temperature of 400 °C. To this regard, XRD still indicates comparably broad Bragg reflections after heating (ESI Fig. S7†). Here, it should be noticed that no Bragg reflections of the Pt nanoparticles occur due to their low concentration and small size. Sorption analysis indicates a single drop of the surface area by about 5% to 107 m^2^ g^−1^ while the pore volume remains constant at 0.082 cm^3^ g^−1^ after heating to 400 °C for 24 h. For longer heating, both surface area and pore volume remain stable. Furthermore, STEM images of the Pt–CeO_2_ nanosponges point to the stability of the inner pore structure of the nanosponges as well as the stability of the size and distribution of the Pt nanoparticles. Thus, the structure and porosity of the CeO_2_ framework as well as a size and distribution of the Pt nanoparticles for the samples heated to 400 °C (Fig. 6) are quite similar to the as-prepared Pt–CeO_2_ nanosponges (Fig. 5).

### Oxidation catalysis with Pt–CeO_2_ nanosponges

To evaluate the potential of the novel Pt–CeO_2_ nanosponges for oxidation catalysis, different reactants and reactions were tested, using standard conditions (*e.g.*, applied temperature, pressure, concentration) without a further optimization of the nanosponge catalyst and/or the respective conditions of the reaction. Specifically, the catalytic oxidation of CO, volatile organic compounds (VOCs) and NH_3_ were studied with regard to activity and temperature range up to 250 °C for CO oxidation and 400 °C for NH_3_ oxidation. As a representative of VOCs, the oxidation of formaldehyde (HCHO) was investigated up to temperature of 250 °C.

**Fig. 7:**
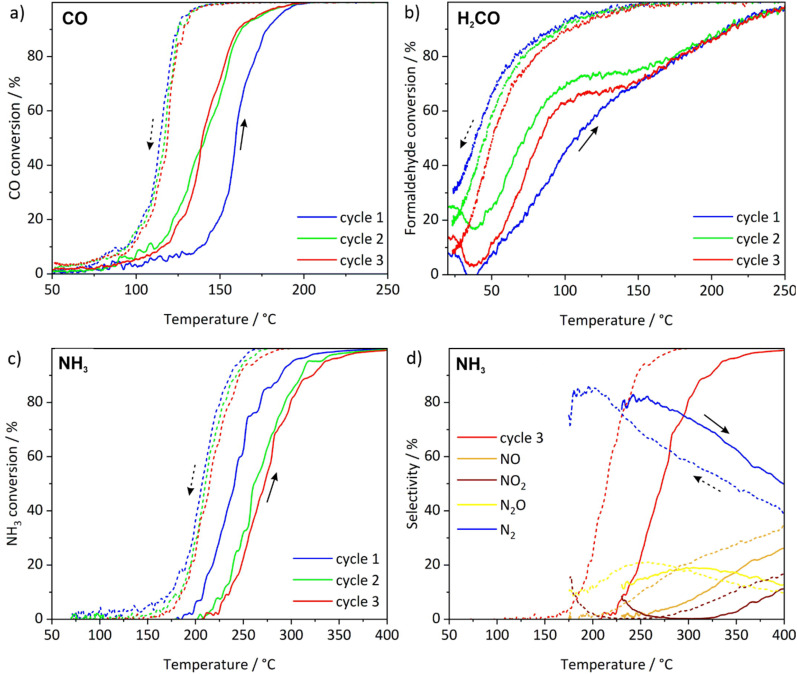
Evaluation of the catalytic properties of the Pt–CeO_2_ (1%) nanosponges: (a) CO oxidation (gas mixture: 50 mL min^−1^ of 1000 ppm CO, 10% O_2_ in He, temperature ramp rate: 5 °C min^−1^), (b) VOC oxidation (gas mixture: 50 mL min^−1^ of 260 ppm HCHO, 10% O_2_ in He, temperature ramp rate: 5 °C min^−1^), (c) NH_3_ oxidation (gas mixture: 50 mL min^−1^ of 1000 ppm CO, 10% O_2_ in He, temperature ramp rate: 10 °C min^−1^), (d) NH_3_ oxidation product selectivity at cycle 3 (for conversion >10%).

The CO, HCHO and NH_3_ conversion and the catalyst stability were monitored in three single experiments with three consecutive light-off/light-out cycles, while applying a ramp rate of 5 °C min^−1^ for CO and HCHO oxidation as well as a ramp rate of 10 °C min^−1^ for NH_3_ oxidation (Fig. 7). In this way, not only the initial activity was determined but also the performance after catalyst degreening under reaction conditions. When considering that the Pt–CeO_2_ nanosponges were exposed to ambient atmosphere prior to the catalytic tests, at least a partial oxidation of the Pt nanoparticle surface is to be expected. As a result, the CO, HCHO, and NH_3_ oxidation is anticipated to occur only at temperatures sufficiently high for the reduction of Pt nanoparticles under reaction conditions.^23^ A relatively high light-off temperature – the temperature at which 50% of activity were reached – of 159 °C during the heating phase, and a slow increase of the CO oxidation activity were observed for the 1st cycle (Fig. 7a). In contrast, 100% conversion was maintained during the cooling down step even at 135 °C, followed by a sharp decrease in activity and a light-out temperature of 114 °C (the temperature at which 50% activity are reached in the cooling cycle, Table 2). Despite the reducing treatment (N_2_ : H_2_ = 10 : 90) applied at 25 °C for the sample, this behaviour can be ascribed to a certain catalyst degreening, including the removal of precursor traces and changes in the oxidation state during the reaction.^24^ Additionally, the noble metal structure is expected to change under reaction conditions due to the interaction with the CeO_2_ support, leading to sintering/redispersion processes depending on the temperature and CO concentration.^5*a*^ The improved activity and the steep CO oxidation profile is then maintained during the 2nd and 3rd reaction cycle. In comparison to the 1st cycle, the light-off and light-out temperatures are very close in values (140 °C and 118 °C, Table 2), showing a good stability of the catalyst system (Fig. 7a). The hysteresis occurring between the light-off and light-out curves points to Pt particles with a size of 1–3 nm as previously reported for alumina-supported catalysts.^24^ This size is also in accordance with STEM images of the Pt–CeO_2_ nanosponges with Pt nanoparticles, 1.8 ± 0.4 nm in size (ESI Fig. S6†). In addition, the different CO oxidation mechanism involving perimeter sites at the interface between the noble metal and the CeO_2_ support can contribute as well to the variation between the heating and cooling curves.^23^ Thus, Ce^3+^ sites are generated at the interface between Pt and CeO_2_ support and in the topmost layer of ceria during CO oxidation.^25^ This availability of oxygen at the perimeter sites further minimizes the CO self-inhibition effect on Pt.^24,26^

**Table 2:** Light-off (*L*_off_) and light-out (*L*_out_) temperatures (indicating a level of 50% activity, *T*_50_, reached during heating or cooling, respectively) for the Pt–CeO_2_ (1%) nanosponges

	1st cycle *L*_off_*T*_50_/°C	1st cycle *L*_out_*T*_50_/°C	2nd cycle *L*_off_*T*_50_/°C	2nd cycle *L*_out_*T*_50_/°C	3rd cycle *L*_off_*T*_50_/°C	3rd cycle *L*_out_*T*_50_/°C
CO	159	114	141	117	140	119
HCHO (VOCs)	107	39	73	46	83	51
NH_3_	241	207	261	210	272	215

A comparable behaviour as for the catalytic CO oxidation with Pt–CeO_2_ nanosponges is also observed for the 1st cycle of the catalytic HCHO oxidation with a light-off temperature of 107 °C but in combination with a very low light-out temperature of 39 °C (Fig. 7b). For the 2nd and 3rd cycle, the difference between light-off and light-out temperature becomes much smaller reaching 83 and 51 °C for the 3rd cycle (Table 2). For the catalytic NH_3_ oxidation, the Pt–CeO_2_ nanosponges show a light-off temperature of 241 °C and a light-out temperature of 207 °C in the 1st cycle (Fig. 7c), which are shifted after the 1st cycle to stable values in the 2nd and 3rd catalytic cycle. Due to the lower reactivity of NH_3_, both the light-off and light-out temperatures are higher compared to CO and HCHO oxidation (Table 2). Finally, the high selectivity of the Pt–CeO_2_ nanosponges for catalytic NH_3_ oxidation is remarkable with >80% of N_2_ at 220–250 °C (light off of 3rd cycle) and 170–200 °C (light out of 3rd cycle) (Fig. 7d).^27^ The selectivity to nitrogen is high up to 300 °C. At higher temperatures, both NO and NO_2_ are formed due to over-oxidation. Compared to the activity reported in the literature for small platinum species on non-interacting support, the Pt–CeO_2_ nanosponges show a high activity with comparable if not better selectivity to nitrogen and low selectivity towards N_2_O. The CeO_2_ nanosponges appears to significantly support the reaction.^27^

Although a reliable comparison with industrially applied catalyst systems or other Pt–CeO_2_ catalysts reported in the literature is difficult due to different types of materials and conditions, the novel Pt–CeO_2_ nanosponges show very promising catalytic performance regarding the oxidation of CO, HCHO, and NH_3_. This holds the more as the nanosponges were not yet optimized in regard of their surface area, pore size and volume, the Pt load and particle size or the specific conditions of the catalytic reaction.

## Conclusions

Pt–CeO_2_ nanosponges with high surface area (113 m^2^ g^−1^), pore volume (0.08 cm^3^ g^−1^), small-sized Pt nanoparticles (1.8 ± 0.4 nm), and good thermal stability (up to 400 °C) are obtained by an optimised thermal decomposition of Ce_2_(C_2_O_4_)_3_·10H_2_O precursor particles. The latter are prepared by precipitation upon reaction of Ce(NO_3_)_3_·6H_2_O and K_2_C_2_O_4_·H_2_O in an ethanol–water mixture. The cerium oxalate precursor is then thermally converted to CeO_2_ nanosponges by heating to 350 °C in air (20 min). Finally, the CeO_2_ nanosponges are decorated with small-sized Pt nanoparticles using a wet-chemical process with Pt(ac)_2_ in methanol. The resulting Pt–CeO_2_ nanosponges (1 wt% Pt) show promising activity for the catalytic oxidation of CO, volatile organic compounds (VOCs), and NH_3_ with low light-out and light-off temperatures, and – in the case of the NH_3_ oxidation – good selectivity for N_2_ formation (>80% at 170–250 °C). Although the Pt–CeO_2_ nanosponges have not yet been optimized in detail, *e.g.*, with respect to pore size and volume, the Pt loading and particle size or the specific conditions of the catalytic reactions, a further increase of the performance can be expected. The synthesis strategy using metal oxalates as precursors for high-surface-area metal oxides in the low-temperature conversion regime can be transferred also to other metal oxides. New material and catalyst concepts with promising results, finally, might also be transferable to other oxides and catalysts, supplemented by further screening in catalyst composition (*e.g.*, variation in noble metal loading, testing under more realistic conditions, long-term durability tests).

## Experimental section

### General

Ce(NO_3_)_3_·6H_2_O (extra pure, Merck), ethanol (99.9%, Seulberger), methanol (99.9%, Seulberger) K_2_C_2_O_4_·H_2_O (P. A., Merck) were handled as purchased. Pt(Ac)_2_ was prepared according to the literature.^28^

### Ce_2_(C_2_O_4_)_3_·10 H_2_O precursor particles

631 mg of Ce(NO_3_)_3_·6H_2_O were dissolved in 150 mL of ethanol. In addition, 442 mg of K_2_C_2_O_4_·H_2_O were dissolved in 15 mL of water. This latter solution was injected with intense stirring at room temperature to the Ce(NO_3_)_3_·6H_2_O solution. The immediate nucleation of the precursor particles was indicated by the formation of a suspension, which was stirred over night to yield crystalline Ce_2_(C_2_O_4_)_3_·10H_2_O. The as-prepared Ce_2_(C_2_O_4_)_3_·10H_2_O precursor particles were purified by centrifugation/resuspension twice from/in water. Finally, the Ce_2_(C_2_O_4_)_3_·10H_2_O precursor particles were dried at 70 °C over night.

### Thermal conversion of precursor particles to CeO_2_ nanosponges

The thermal conversion of the Ce_2_(C_2_O_4_)_3_·10H_2_O precursor particles to the CeO_2_ nanosponges with optimal performance was performed by heating at 350 °C for 20 min. Thereafter, the CeO_2_ nanosponges were redispersed/centrifuged twice in/from water to remove all remaining KNO_3_. Finally, the CeO_2_ nanosponges were dried at 70 °C over night.

### Modification with Pt nanoparticles

The CeO_2_ nanosponges were impregnated with Pt (1 wt%) by a wet-chemical process. To this concern, 4.2 mg of Pt(ac)_2_ were dissolved in 0.3 mL of methanol and dropped on 200 mg of a dried powder sample of the CeO_2_ nanosponges. This was conducted in two steps with addition of 0.15 mL for each and drying at 70 °C after each step. To obtain Pt nanoparticles, dried powder samples were treated in reducing-gas atmosphere (N_2_ : H_2_ = 90 : 10) for 30 min at room temperature. The formation of Pt nanoparticles can be followed with the naked eye due to the color change from greenish-blue to grey.

### CO oxidation

Sieved Pt–CeO_2_ nanosponge powders (5 mg, 125–250 μm grain size) were placed in quartz microreactors (*Ø*: 1.5 mm) with quartz-wool plugs in the front and at the back of the catalyst bed. A gas mixture of 50 mL min^−1^ with 1000 ppm CO and 10% O_2_ in He was dosed over that catalyst bed at ambient pressure. This corresponds to weight hourly space velocity (WHSV) of 30 000 L g_Pt_^−1^ h^−1^. The temperature was varied between 50–250 °C with a heating/cooling rate of 5 °C min^−1^. The outlet-gas composition was analyzed with an FT-IR spectrometer (Multigas 2030 Analyzer™, MKS Instruments) with a focus on the CO and CO_2_ concentration.

### NH_3_ oxidation

For ammonia oxidation similar reaction conditions were used. Analogously, sieved Pt–CeO_2_ nanosponge powders (5 mg, 125–250 μm grain size) were used (quartz microreactors with *Ø*: 1.5 mm, WHSV of 30 000 L g_Pt_^−1^ h^−1^, FT-IR analysis with same analyzer as for CO-oxidation). For the experiments, a gas mixture of 50 mL min^−1^ with 1000 ppm NH_3_ and 10% O_2_ in He was dosed between 50–400 °C (heating/cooling rate of 10 °C min^−1^). The NH_3_ concentration together with that of possible reaction products (*i.e.*, NO, N_2_O, NO_2_) was determined.

### Formaldehyde oxidation

Analogously, sieved Pt–CeO_2_ nanosponge powders (5 mg, 125–250 μm grain size) were placed in the same quartz microreactors (*Ø*: 1.5 mm) and fixed in place using quartz-wool plugs. For the experiments, a gas mixture of 50 mL min^−1^ with 260 ppm formaldehyde and 10% O_2_ in He was dosed over that catalyst bed at ambient pressure (WHSV of 30 000 L g_Pt_^−1^ h^−1^). Formaldehyde was dosed using a gas saturator containing a commercial 16% formaldehyde solution in water (Science Services). The temperature was varied between 25–250 °C with a heating/cooling rate of 5 °C min^−1^. The outlet gas composition was analyzed by FT-IR determining the concentration of HCHO together with the possible reaction products (CO and CO_2_). Further details regarding the analytical equipment can be obtained from the ESI.†

## Data availability

Additional data regarding experiments and methods can be obtained from the ESI† and on request from the authors.

## Conflicts of interest

The authors declare no competing financial interests.

## Supplementary Material

NA-OLF-D4NA00525B-s001

NA-OLF-D4NA00525B-s002

NA-OLF-D4NA00525B-s003
