# Supplementary material for: High-porosity Pt–CeO2 nanosponges as oxidation catalyst
Source: Nanoscale Adv. 2024 Dec 30;7(4):1173–82. doi: 10.1039/d4na00525b (PMC11705977; doi:10.1039/d4na00525b)
Supplement: NA-007-D4NA00525B-s001 [file NA-007-D4NA00525B-s001.pdf]

Supplementary Information (SI) for Nanoscale Advances.
This journal is © The Royal Society of Chemistry 2024

High-Porosity Pt-CeO_2 Nanosponges as Oxidation Catalyst

Simon Falkner[1], Carina B. Maliakkal[2], Mareike Liebertseder[1], Joachim Czechowsky[3], Maria Casapu[3], Jan-Dierk Grunwaldt[3,4], Christian Kübel[2], and Claus Feldmann[1]**

[1] MSc. S. . Falkner, Dr. M. Liebertseder, Prof. Dr. C. Feldmann
Institute of Inorganic Chemistry (IAC), Karlsruhe Institute of Technology (KIT)
Engesserstraße 15, D-76131 Karlsruhe (Germany)
E-mail: claus.feldmann@kit.edu

[2] Dr. C. B. Maliakkal, Prof. Dr. C. Kübel
Institute of Nanotechnology (INT) and Karlsruhe Nano Micro Facility (KNMFi), Karlsruhe Institute of Technology (KIT), Hermann-von-Helmholtz-Platz 1, 76344 Eggenstein-Leopoldshafen (Germany)

[3] MSc. Joachim Czechowsky, Dr. M. Casapu, Prof. Dr. J.-D. Grunwaldt
Institute for Chemical Technology and Polymer Chemistry (ITCP), Karlsruhe Institute of Technology (KIT), Engesserstraße 20, 76131 Karlsruhe (Germany)

[4] Prof. Dr. J.-D. Grunwaldt
Institute of Catalysis Research and Technology (IKFT), Karlsruher Institute of Technology (KIT), Hermann-von-Helmholtz-Platz 1, 76344 Eggenstein-Leopoldshafen (Germany)

# – Supporting Information –

Content:
1. Analytical Techniques
2. Material Characterization

1. Analytical Techniques

Transmission electron microscopy (TEM). TEM was conducted on a FEI Osiris microscope operated at 200 kV in STEM mode, a probe corrected Thermo Fischer Scientific (TFS) Themis 300 operated in STEM mode at 300 kV, and a double corrected TFS Themis Z operated in STEM or TEM mode at 300 kV. The Osiris microscope was equipped with a Bruker Quantax system (XFlash detector) and the Themis Z/300 with a Super-X EDX detector. EDX spectra were quantified with the TFS software package "TEM imaging and analysis" (TIA) and Velox (version 3.9). TEM samples were prepared by putting a drop of suspension of the respective powder sample in ethanol onto a copper grid coated with amorphous carbon (Lacey-) supporting film or by dry shaking the TEM grid in the powder catalyst. Average particle diameters were calculated by statistical evaluation of at least 150 nanoparticles (ImageJ 1.47v software). Electron tomography was utilised to determine the 3-dimensional structure of the ceria nanosponges. Series of STEM images for tomography were acquired with a tilt interval of 2° using the TFS STEM tomography software. The tilt angle range was from –74° to +76° for the nanosponge shown in the *main paper: Figure 4d*, and -66° to +70° for the nanosponge shown in the *main paper: Figure 4f.* Alignment of the tilt-series was done using ETomo/Imod 4.7 software with residual error below 0.4 pixel. The reconstruction of the 3D volume was done using the SIRT algorithm implemented in Inspect3D V4.4 software. 3D visualization was done in the Avizo software 2021.1.

X-ray powder diffraction (XRD). X-ray powder diffraction (XRD) was performed with a Stoe STADI-MP diffractometer operating with Ge-monochromatized Cu-$K\alpha$-radiation (λ = 1.54178 Å) and Debye-Scherer geometry. The dried samples were fixed between Scotch tape and acetate paper and measured between -69° and +69° of two-theta.

Volumetric sorption analysis was carried out with an AUTOSORB IQ-XR VITON (Anton Paar), applying N_2 as adsorbate. The specific surface area was determined using the Brunauer-Emmett-Teller (BET) theory. The pore analysis was performed using DFT-based calculations.

2. Material Characterization

Sorption data with adsorption isotherms and desorption isotherms, using nitrogen as the sorbent, as well as pore volume and pore diameter as obtained via DFT-based calculations[S1] are shown for $Ce_2(C_2O_4)_3 \cdot 10\ H_2O$ precursor particles (Figure S1), CeO_2 nanosponges (Figure S2), and Pt-CeO_2 nanosponges (Figure S3). The CeO_2 nanosponges and the Pt-decorated CeO_2 nanosponges are more-or-less identical.

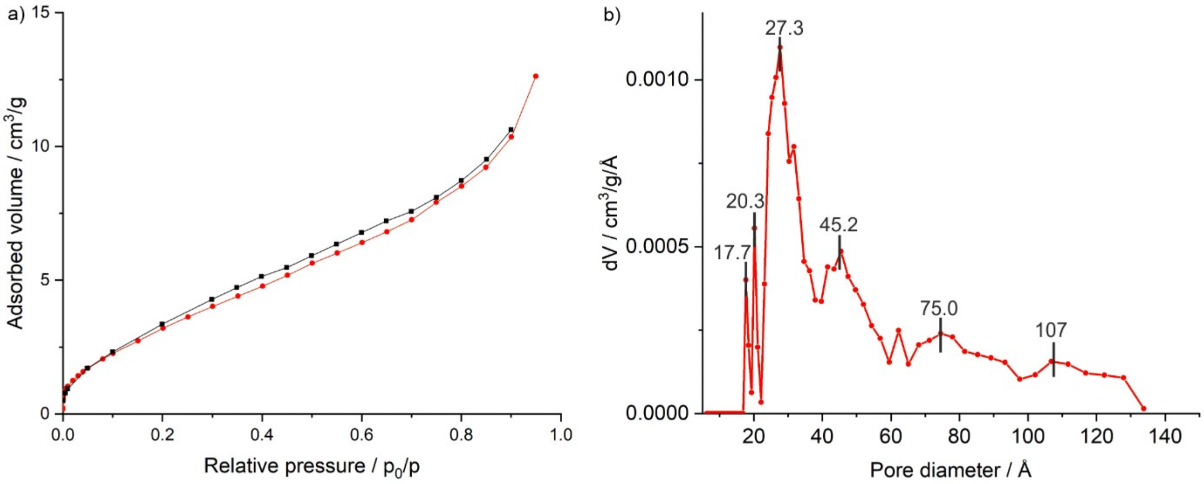

Figure S1. Sorption data of the $Ce_2(C_2O_4)_3 \cdot 10\ H_2O$ precursor particles: a) adsorption isotherm (red) and desorption isotherm (black) with nitrogen as the sorbent, b) evaluation of sorption data via DFT-based calculations to obtain pore volume and pore diameter.

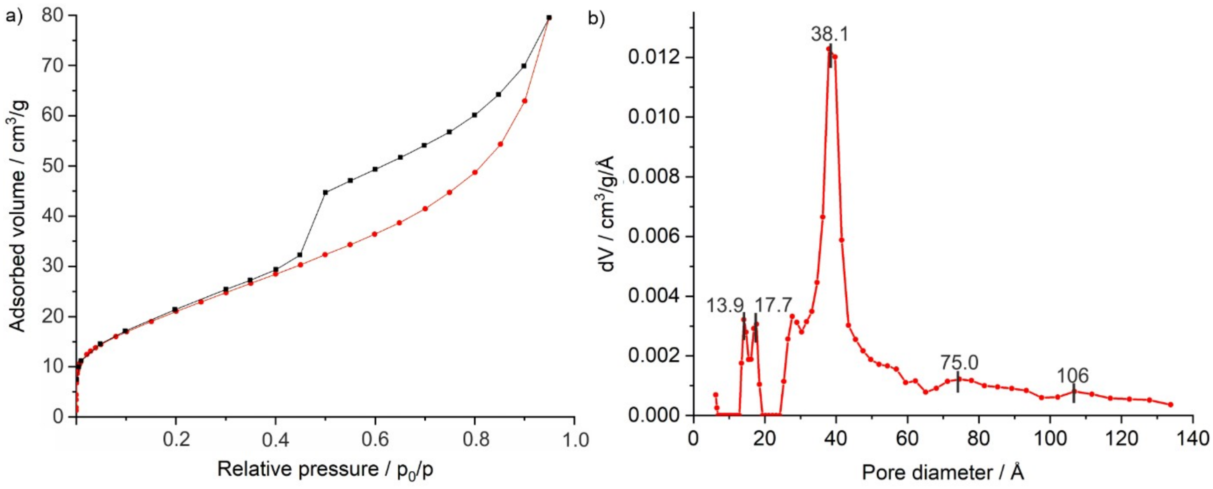

Figure S2. Sorption data of the CeO_2 nanosponges: a) adsorption isotherm (red) and desorption isotherm (black) with nitrogen as the sorbent, b) evaluation of sorption data via DFT-based calculations to obtain pore volume and pore diameter.

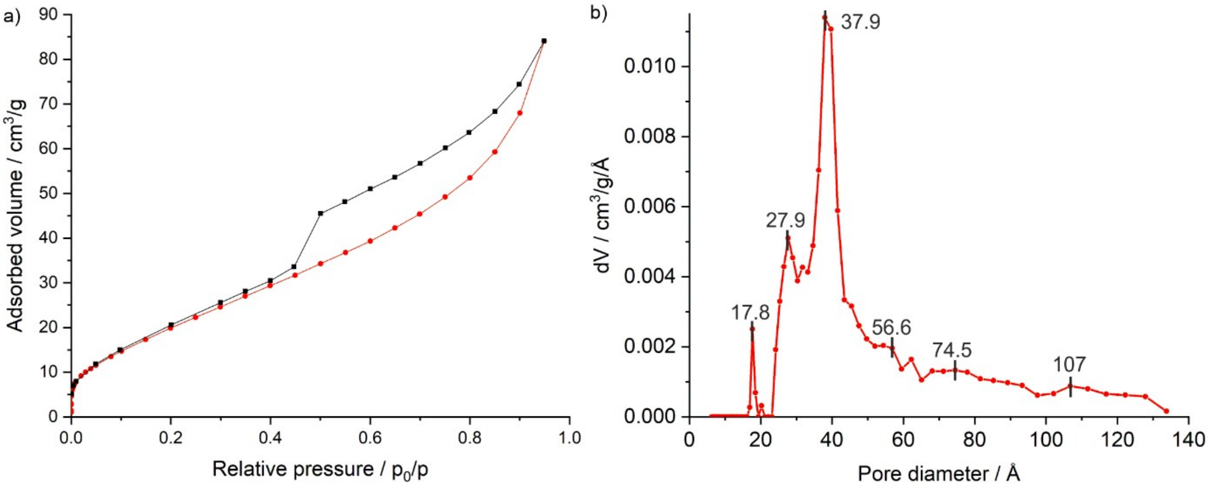

Figure S3. Sorption data of the Pt-CeO_2 nanosponges: a) adsorption isotherm (red) and desorption isotherm (black) with nitrogen as the sorbent, b) evaluation of sorption data via DFT-based calculations to obtain pore volume and pore diameter.

According to electron diffraction, the CeO_2 nanosponges are highly crystalline (Figure S4), which confirms the results from X-ray powder diffraction (*see main paper: Figure 3d*). The diffraction pattern was indexed to the CaF_2 structure of ceria.

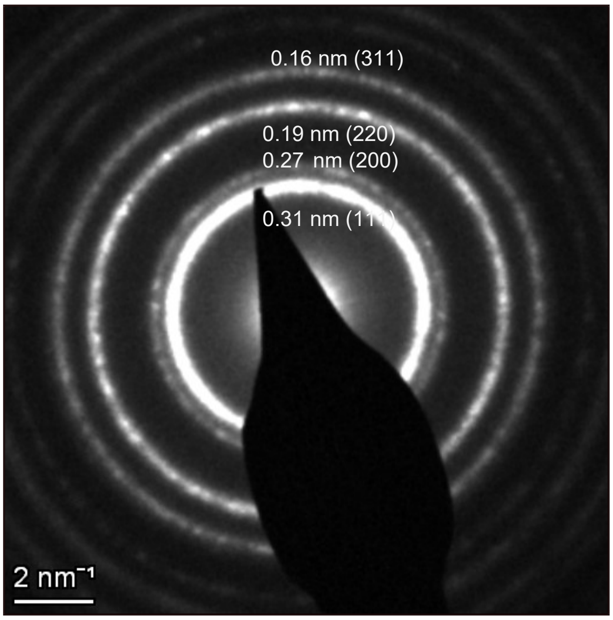

Figure S4. Diffraction pattern (*inset in main paper: Figure 4c*) with marked lattice spacing and crystal planes.

The porosity of the CeO_2 nanosponges is evidenced by electron microscopy and tomography (*see main paper: Figure 4*). Volume rendering and reconstruction of Z-slices of the CeO_2 nanosponges are also illustrated in Figure S5 and the Videos S1-S4.

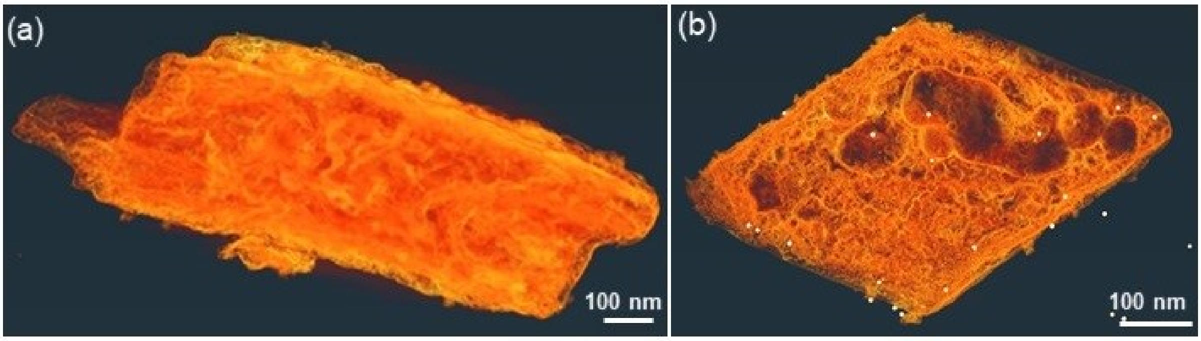

Figure S5. Volume rendering and reconstruction of Z-slices of the CeO_2 nanosponges: a) and b) show volume rendering based on electron tomography of the CeO_2 nanosponges (*see main paper: Figure 4d,f*). For volume rendering and reconstruction of Z-slices also see the Videos S1-S4).

The size distribution of the Pt nanoparticles on the Pt-CeO_2 nanosponges was deduced from > 200 Pt nanoparticles on HAADF-STEM images to a mean value of 1.8±0.4 nm (Figure S6).

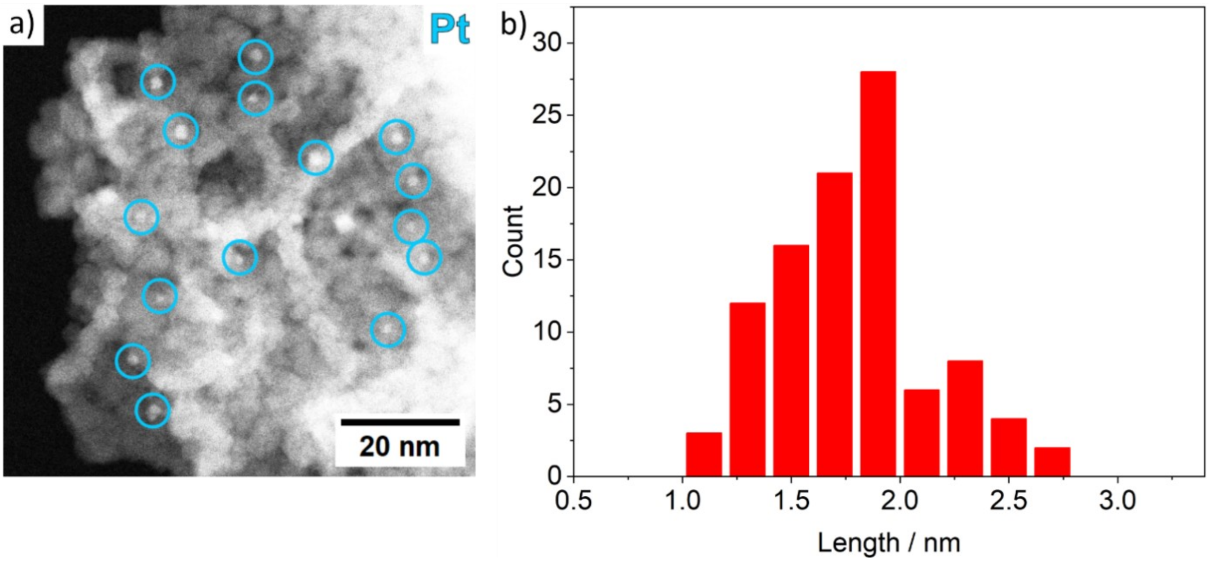

Figure S6. Size distribution of the Pt nanoparticles on Pt-CeO_2 nanosponges: a) HAADF-STEM image (with some single Pt nanoparticles marked with blue circles), b) size distribution of the Pt nanoparticles.

The thermal stability of the Pt-CeO_2 nanosponges was examined by sorption analysis and TEM up to a temperature of 400 °C (*see main paper: Figure 6*). Moreover, XRD shows Bragg reflections that are still broad and similar to the as-prepared CeO_2 nanosponges (Figure S7).

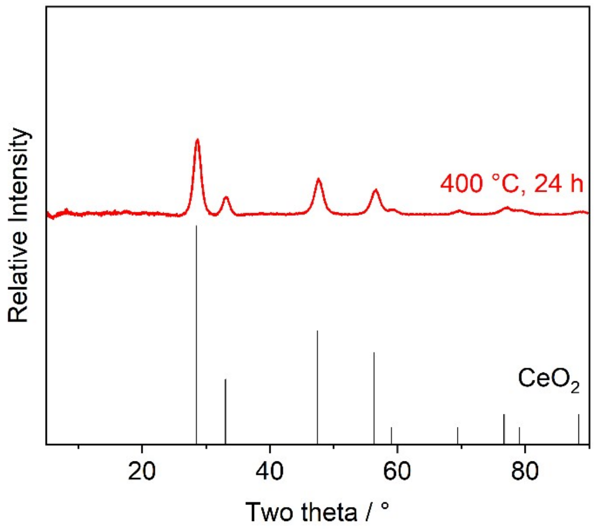

Figure S7. XRD of Pt-CeO_2 nanosponges after heating to 400 °C for 24 hours (CeO_2 as a reference, ICDD-No. C01-075-7101).

References

S1 A. Galarneau, F. Villemot, J. Rodriguez, F. Fajula and B. Coasne, *Langmuir*, **2014**, *30*, 13266-13274.
