# Supplementary figures and images for: High-porosity Pt–CeO2 nanosponges as oxidation catalyst
Source: Nanoscale Adv. 2024 Dec 30;7(4):1173–82. doi: 10.1039/d4na00525b (PMC11705977; doi:10.1039/d4na00525b)

## Videos S1+S2 (associated with Figure 4d,e)

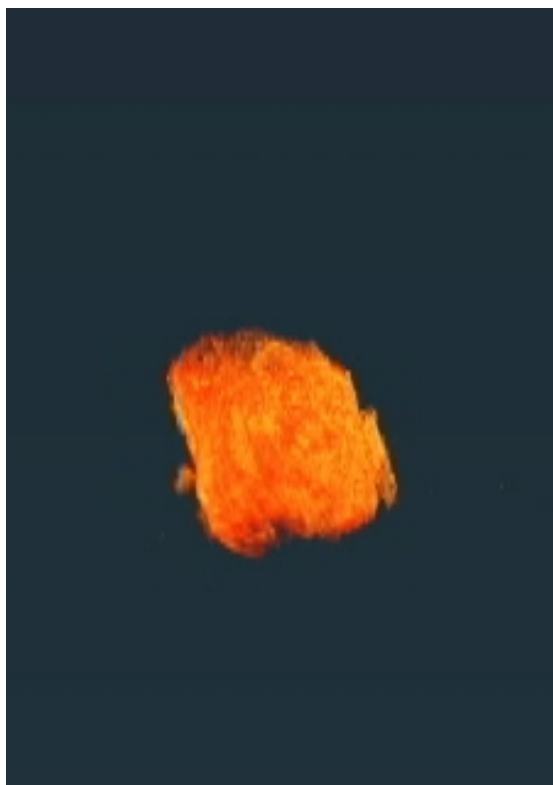

Volume rendering video

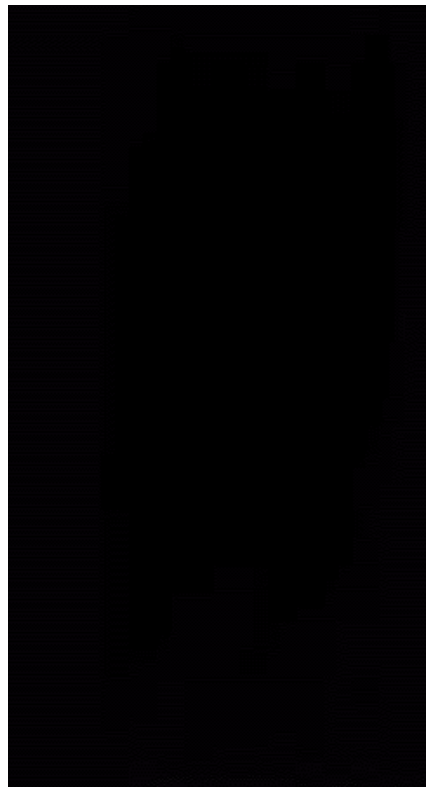

Reconstruction: Z slices video

Supplement: NA-007-D4NA00525B-s002 [file NA-007-D4NA00525B-s002.pdf]

## Video S3+S4 (associated with Figure 4f,g)

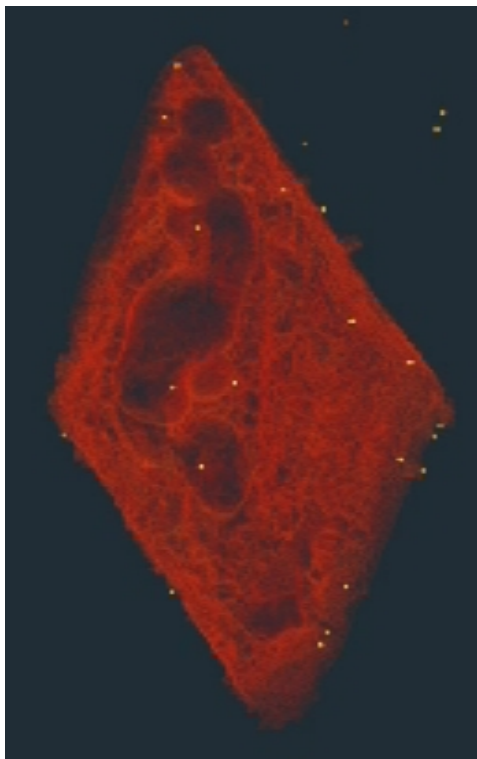

Volume rendering video

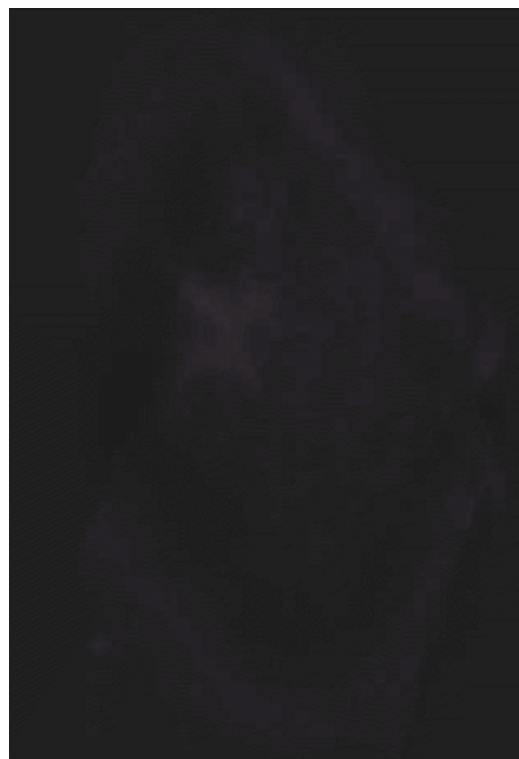

Reconstruction: Z slices video

Supplement: NA-007-D4NA00525B-s003 [file NA-007-D4NA00525B-s003.pdf]
